# A Great and Commendable Work

**Published:** 1912-01

**Authors:** James M. Lynch

**Affiliations:** President; Indianapolis, Indiana


### Abstracts and Selections.

### A Great and Commendable Work.

[We publish below a circular letter concerning the work of the printers in collaboration with the medical profession in the war against tuberculosis.—Ed.]

INDIANAPOLIS, INDIANA.

DEAR SIR: To you as a citizen and as a student of economic conditions, this letter is addressed. Trade unionism has been much in the public eye for several years, and the effort of the wage earner to better his position, both as to wages earned and working conditions, has received the approbation or the disapproval of the citizen, often without investigation or any accurate knowledge as to the cause of the strife that has often followed the attempt referred to. It seems to me that our critics should study the trade unions, their activities and beneficences, their aims and ideals, the obstacles that confront them and conditions that surround them, and it is my belief that if this is done it can but be of benefit to the trade unions.

One of the beneficences of the International Typographical Union is its home at Colorado Springs—the Union Printers' Home, erected and maintained by the International Typographical Union, the only institution of its kind in the world. It was formally dedicated in 1892, and since its doors were opened for the reception of the aged and sick the International Typographical Union has expended about one million dollars in its maintenance.

The physical value of the property is about one million dollars.

Originally there was one building; now there are a number of buildings. The home cares for the aged members of the I. T. U. and for its sick and distressed.

The home furnishes the resident without cost everything he needs—food, shelter and clothing. In addition, 50 cents a week is allowed for incidentals that may be desired or required by the resident. The home's charity is unpurchasable, its bounty without price.

Since its institution, and especially since the establishment of the tuberculosis sanatorium and the segregation of tuberculosis patients, a large number of our members have been cured at the home, have returned to work and are now self-supporting and useful members of society.

In addition to the home we have a pension system under which any member of twenty years' standing, sixty years or more of age and who is unable to obtain sustaining employment at the compositor's trade, is paid a pension of $5.00 per week for the balance of his life. Any member who has a continuous membership of twenty years and whose application for admission to the home has been rejected because of certain diseases that are contagious, is also paid a pension of $5.00 per week.

It is the boast of the International Typographical Union that its members do not become public charges; that they are not to be found in almshouses or workhouses.

The International Typographical Union of North America is an organization built upon the most democratic principles—all constitutional changes and enactments increasing taxation being submitted to the members and approved by a majority before becoming laws. Thus the governing and taxing power is properly placed—in the hands of the members.

Some of its aims:

To elevate the position and maintain and protect the interests of the craft in general.

To establish and uphold a fair and equitable rate of wages, and to regulate all trade matters appertaining to the welfare of members.

To influence the apprenticeship system in the direction of intelligence, competency and skill, in the interest alike of employer and employe.

To endeavor to replace strikes and their attendant bitterness and pecuniary loss by arbitration and conciliation in the settlement of all disputes concerning wages and conditions of employment.

To relieve the deserving needy, and provide for the proper burial of deceased members.

If at any time you contemplate a trip to Colorado, be sure and include the Union Printers' Home in your sight-seeing itinerary. The home is open for inspection from 9 a. m. to 5 p. m. Guides are furnished the visitors, and we are pleased to have them inspect the institution.

A word from you as to this phase of the activities of the International Typographical Union will be appreciated.

It also will be appreciated (if you approve of what the union printers of the country are doing) if you will send your orders for printing to offices employing the members of the I. T. U.

Both as to the pension, as to the mortuary benefit, as to technical education for journeymen and apprentices, and as to our home, we believe we are doing a great work, but we can only continue this work under fair conditions as to wages and hours, and these obtain in union printing offices.

Sincerely,

JAMES M. LYNCH,

President.

